# Building Specialized Nursing Practice Capacity in Bangladesh: An Educational Program to Prepare Nurses to Care for Oncology and Bone Marrow Transplant Patients in Dhaka, Bangladesh

**DOI:** 10.1200/JGO.2016.006486

**Published:** 2016-12-21

**Authors:** Anne-Marie Barron, Jenna Moran, Shabnam Sultana Nina, Jason Harlow, Meena Gyawali, Farhad Hossain, Mark Brezina, Caroline Callahan, Judy Curran, Colleen Danielson, Ellen Fitzgerald, Judy Foster, Emily Erhardt, Christine Shaughnessy, Albert C. Yeh, Bimalangshu R. Dey

**Affiliations:** **Anne-Marie Barron,** Simmons College School of Nursing and Health Science; **Jenna Moran,** **Mark Brezina,** **Caroline Callahan,** **Judy Curran,** **Colleen Danielson,** **Ellen Fitzgerald,** **Judy Foster,** **Emily Erhardt,** **Christine Shaughnessy,** and **Albert C. Yeh,** Massachusetts General Hospital; **Jenna Moran,** **Mark Brezina,** **Caroline Callahan,** **Judy Curran,** **Colleen Danielson,** **Ellen Fitzgerald,** **Judy Foster,** and **Bimalangshu R. Dey,** Massachusetts General Hospital Bone Marrow Transplant Program; **Jason Harlow,** Massachusetts General Hospital Center for Global Health; **Bimalangshu R. Dey,** Massachusetts General Hospital Cancer Center, Boston, MA; and **Shabnam Sultana Nina,** **Meena Gyawali,** and **Farhad Hossain,** AK Khan Healthcare Trust, Dhaka, Bangladesh.

## Abstract

In 2012, the Minister of Health and other leaders in the Bangladesh government approached Massachusetts General Hospital to establish the country’s first bone marrow transplant program at Dhaka Medical College Hospital to serve the needs of the people of Bangladesh. Stated goals of this collaboration included a broad focus on the care of oncology patients with a specific emphasis on care of patients with hematologic malignancies and of women with gynecologic cancers. The purpose of this article is to describe the international nursing collaboration between Massachusetts General Hospital, Simmons College, the AK Khan Healthcare Trust in Dhaka, and Dhaka Medical College Hospital that was established to share nursing knowledge and to build specialized professional nursing capacities to deliver high-quality cancer care in the public sector. Over the past 3 years, through the educational programs that have been developed within this collaboration—the Enhanced Specialized Nurse Training Program—the Bangladeshi nurses have received continuing professional development based on Western standards of nursing and have been offering nursing care to patients who have undergone chemotherapy and bone marrow transplantation. The challenges, opportunities, and outcomes of this international collaboration have been highly rewarding and mutually beneficial.

## INTRODUCTION

The people of Bangladesh have suffered from poverty, overcrowding, and lack of health care access at all levels.^1^ Whereas considerable gains have been made in the areas of population control, life expectancy, and maternal and neonatal death rates, significant health-related concerns remain.^2^ Noncommunicable diseases, including cancer, heart disease, stroke, hypertension, diabetes, and chronic respiratory disease, represent a significant and rising share of the global disease burden and account for more than one half of all deaths in Bangladesh. Effective, integrated management of chronic diseases in Bangladesh requires a sustained effort to increase the capacity, clinical competence, and professional status of nurses. Well-educated nurses with a professional focus on the care of patients within the public sector—where > 90% of the population receive their care—will contribute significantly to the health of the people of Bangladesh and sustain and expand the impressive health gains that have been made in recent years.

The purpose of this article is to describe the collaboration that has been established for the Enhanced Specialized Nurse Training Program (ESNTP) and to serve as a model for elevating nursing education and practice in the developing world. The ENSTP was created in partnership with Dhaka Medical College Hospital (DMCH), health care providers at Massachusetts General Hospital (MGH), the Ministry of Health and Family Welfare, the Government of Bangladesh, the AK Khan Healthcare Trust, and Simmons College as an essential aspect of the creation of the first bone marrow transplant (BMT) program in Bangladesh.

## CANCER BURDEN IN BANGLADESH

According to the National Cancer Control Strategy and Plan of Action 2009 to 2015, cancer is a high-priority focus, in part because of its direct economic impact. Most patients with cancer (66%) are of working age and are routinely and prematurely lost from the nation’s workforce.^3^ It is estimated that Bangladesh currently has approximately 4 million patients with cancer and between 200,000 and 800,000 new cancer diagnoses every year.^4^ Overall population projections estimate that cancer was the main cause in 7.5% of deaths in Bangladesh in 2005, and cancer is projected to constitute 12.7% of total deaths by 2030.^3^ The recently conducted Bangladesh Maternal Mortality and Health Care Survey^5^ estimated that cancer remains the leading cause of death among women of reproductive age, accounting for 21% of total deaths.^6^ The current and projected impact of cancer on the citizens and economy of Bangladesh underscores the dire need for nurses who are well prepared to offer the complex and comprehensive care that patients with cancer and their families require and deserve.

## CHALLENGES OF NURSING IN BANGLADESH

Three important and related factors contribute to the lack of access to quality nursing care in Bangladesh: the low number of practicing nurses; the lack of appropriately skilled nurses, largely as a result of the lack of well-prepared nursing faculty; and the poor image and reputation of nurses in Bangladesh.^7-11^ A major challenge to advancing the profession of nursing in Bangladesh is the severe shortage of nurses and nursing faculty. The WHO (2006) recommends one registered nurse (RN) for every 500 people or 20 RNs per 10,000. The Directorate of Nursing Services in Bangladesh reports that there are 35,000 RNs in Bangladesh (personal communication, Nelofar Farhad, Directorate of Nursing Services, December 2, 2015). With an estimated population of 169 million in 2015,^12^ Bangladesh averages only one RN per 4,800 people. This severe scarcity threatens the health of the people who receive care in public sector hospitals and forces physicians to assume many caretaking activities for patients that are carried out by nurses in other areas of the world.

The low social perception of nursing in Bangladesh is at variance with perceptions in other parts of the world. The confluence of many factors in Bangladesh, such as low pay, the stigma associated with the physical aspects of care, night-shift work, and the history of young women entering diploma nursing programs at the age of sixteen, have contributed to a dangerous and ill-conceived view of the potential of nursing. Furthermore, there are no programs in Bangladesh that confer masters or doctoral degrees in nursing; thus, the available pool of nursing faculty is limited. Fortunately, Dhaka University College of Nursing is in the process of developing graduate nursing programs to begin to address this important need.

## DEVELOPING SOLUTIONS: THE ESNTP

The ESNTP is a 3-year program focused on the nursing care of patients with cancer in Bangladesh, with a specific emphasis on the care of patients undergoing BMT and general oncology patients. The technological and psychosocial demands of offering excellent care to patients with cancer are among the most significant challenges in nursing practice. The continually evolving treatments require ongoing education over the course of nurses’ careers. Without master’s degree programs to prepare nurses in advanced practice specialties, including oncology nursing, there are few opportunities for nurses to gain the expertise and high-level skills required to offer safe, comprehensive, and excellent nursing care to the people of Bangladesh and their families. Until the vision of the Bangladesh Nursing Council, the Directorate of Nursing Services of the Ministry of Health and Family Welfare, and the WHO is realized, and the basic preparation of nurses in Bangladesh at the bachelor’s of science level is offered by nursing faculty who are educated at the master’s and doctoral degree levels within Bangladesh, the continuing education programs for practicing nurses within such programs as ESNTP will be essential to elevate the skills and knowledge of existing nurses.^13^

The focus of the educational programs in the first year was to enhance the skills of nurses to enable them to provide high-quality care for the first patients in Bangladesh who received autologous BMTs; providing high-quality care for solid tumor oncology patients, including women with gynecologic cancers, was the program’s focus in its second year. The third year, which is ongoing, expands the focus to the care of patients who will receive allogeneic BMTs. Nurse experts in Dhaka and the Boston team based at MGH and Simmons College developed and taught three curriculums: Nursing Care of the Autologous Bone Marrow Transplant Patient, Nursing Care of the Allogeneic Stem Cell Transplant Patient (beginning and ongoing), and Nursing Care of the Solid Tumor Oncology Patient. There were 32 members on the Boston-based team who were focused on the ESNTP: 29 nurses and nurse practitioners (NPs), one physician, one global health administrator, and one pharmacist. Thirty members of the team traveled to DHCH during the first 2 years of the program to deliver and evaluate the BMT and oncology curriculums.

Faculty at the AK Khan Healthcare Trust, a nonprofit organization and local technical partner in Dhaka, developed and delivered the first 4 months of each of the curriculums for the first and second years. They offered intensive English instruction and a review of fundamental nursing skills. The Trust’s faculty consists of a director who is a specialist in teaching English and two nursing faculty with master’s degrees. MGH nursing faculty delivered specialty-specific education and training over a span of 6 months in each of the first and second year projects. The Trust’s faculty facilitated and actively participated in the teaching of the BMT and oncology curriculums, which were delivered on-site by MGH nursing faculty. They offered ongoing support to the Boston-based faculty as they presented the BMT and oncology curriculums in Dhaka, reinforced the content of each curriculum, and supported the nurses in the first and second years.

The ESNTP took place within the larger context of collaborations in Boston and Dhaka. Nurses and NPs worked closely with hematologists, pharmacists, and technologists and technicians at MGH and DMCH. Physicians, nurses, and technologists from DMCH have traveled to MGH for observation and education, and many members of the health care team at MGH have traveled to DMCH.^14^

## MAJOR OBJECTIVES FOR ESNTP

There were four major overarching objectives for the ESNTP. The goal of the first year was to educate practicing nurses at DMCH so that the citizens of Bangladesh who are in need of life-saving BMTs have access to this treatment within their country. During the second year of the pilot, the model was expanded to offer nurses at DMCH and the National Institute of Cancer Research of Bangladesh an educational program that included the care of general oncology patients. The third year, which is current and ongoing, has expanded the BMT focus to the care of patients undergoing allogeneic BMT in preparation for the expansion of the BMT program in the near future. For the third year, nurses who care for patients undergoing autologous BMT participate in the aspects of the curriculum that address allogeneic BMT care. Twenty nurses completed the first year, 26 completed the second year, and 26 new nurses are currently enrolled in the third year. The nursing care necessary to support patients and families through the rigorous and, at times, life-threatening challenges of BMT and oncology care is complex, demanding, and highly sophisticated. The skills and knowledge required to offer safe and effective nursing care are considerable.

The second major objective was developing and sustaining relationships between and within the Boston and Dhaka teams as well as establishing trust and positive working relationships with the leaders at DMCH, including the director, the nursing matron, and the physicians with expertise in hematologic cancers, general medical oncology, and the care of women with cancer. The robust collaborative efforts and outstanding communication between the minister of health and family welfare and the MGH Center for Global Health greatly assisted with establishing trust and good working relationships across different and remote cultures. They worked closely with the director of DMCH and the director of the AK Khan faculty to ensure that the leadership at DMCH, including those who were part of the newly formed BMT unit and across the medical oncology programs, were well informed and involved at all levels of planning the educational programs. They also helped the Boston team to understand and navigate the cultural influences on the programs.

A third important objective was to stay in close touch with the nursing leaders in Bangladesh to keep them well informed of the projects and to seek their guidance and approvals for the teaching being offered. Members of the MGH and AK Khan Trust faculty met regularly with the registrar of the Bangladesh Nursing Council and the Directorate of Nursing Services. These nursing leaders were supportive of this international nursing collaboration.

One of the biggest challenges in the establishment of the DMCH program was to establish nurses as experts in their own right. In developed countries, transplant nurses assume a large degree of responsibility for managing and identifying complications during the peritransplant period, and they play a critical role on the health care team. However, the level of training and the degree of independence that most nurses have in the developing world lags far behind those of their Western counterparts. In Bangladesh, there has historically been a large gap in the hierarchy between physicians and nursing providers, such that nurses play a much more limited role during rounds and are not able to serve effectively as patient advocates. Given the critical role that nurses have in the day-to-day care of the patient undergoing transplantation, a fourth important objective was to establish a culture in which nurses were seen as equal partners with physicians in the delivery of care for patients undergoing BMT.

One important step in addressing this challenge was to increase the nurses’ knowledge and skills in caring for patients undergoing BMT. Another significant strategy to counter the low social perception of Bangladeshi nurses and to elevate the importance of patient-centered teamwork was the role modeling that MGH physicians and nurses offered to their Bangladesh colleagues. An MGH oncologist conducts rounds each day with Dhaka physicians and nurses, either in person, if possible, or via Skype. Rounds always begin by asking nurses for their assessment of each patient and including them in the comprehensive teaching rounds for each patient. The Trust faculty and MGH nurses also guide and support the active participation in rounds and other teaching activities of the DMCH nurses. Nurses’ active engagement in rounds underscores their central roles, both in delivering the sophisticated nursing care that is so important for patients and as essential partners of the patient care team.

Nurses and NPs from MGH are highly regarded in Bangladesh. They actively consult with physicians and nurses when they are in Dhaka. The advanced practice skills of the NPs are especially meaningful to the physicians. NP to MD consultation offers new levels of collaboration between nurses and physicians in Bangladesh. The role modeling of respectful partnership between physicians and nurses offered by the nurses and nurse practitioners from MGH when they are on-site at DMCH is invaluable in demonstrating the power of equal and shared partnership on the outcomes of patient care and on heightened professional satisfaction. Shifting the perception of nurses and recognizing them as equal partners in the delivery of care is a challenging, ongoing process in Bangladesh that represents a major cultural change.

## TEACHING METHODS AND MEASUREMENT OF OUTCOMES

Instructional methods included lectures, case studies, seminar discussions, demonstration and feedback on specific clinical competencies, mentoring on the clinical units, participation in teaching rounds, and educational reinforcement with clinically focused Skype discussions and ongoing lectures. Twenty-seven nurses and NPs delivered the instruction on-site at DMCH during the first 3 years.

Nurse-learner outcomes were measured in a number of ways that evaluated their knowledge and clinical skills. Quizzes and formal written and oral examinations assessed mastery of the content that was presented in the curriculum. Participants successfully demonstrated clinical skill competencies in the classroom, laboratory, and on clinical units. Over the course of all programs, nurse participants presented patient cases to faculty and participant colleagues and prepared formal presentations of key concepts of the curriculum to faculty and peers. On several occasions, they also presented key concepts to medical students and residents. The faculty carefully assessed the nurses’ documentation of patient care in their nursing progress notes.

At the end of the first and second years, nurse participants were intensively evaluated. They successfully completed a 100-question comprehensive final examination and a 20-question oral exam that were based on patient case studies. All of the nurses who participated in the first and second year programs were successful on all measures of competence. The nurses in the current, third year of the BMT program will experience the same evaluation process at the end of their program.

Perhaps the greatest outcome indicator of the nurses’ participation in the ESNTP is reflected in the successful outcome for patients who received transplantations during the first 2 years of the BMT program. As of May 2016, 21 patients (age 18 to 58 years) had undergone autologous transplantations at DMCH. We have treated 11 patients with myeloma, four with diffuse large B-cell lymphoma, four with Hodgkin’s lymphoma, one with acute myelogenous leukemia, and one with peripheral T-cell lymphoma.^14^ Conditioning regimens used included melphalan (11 patients), BEAM (carmustine, etoposide, cytarabine, and melphalan; nine patients), and busulfan plus cyclophosphamide (one patient). There have been no transplant-related mortalities to date. There were 10 documented infections, including seven cases of bacteremia, two *Clostridium difficile* infections, and one case of pneumonia. Five patients have experienced relapse (ranging from day 213 to day 598), and the longest disease-free survivor is now 639 days out from transplantation. Nurses have been instrumental in caring well for these patients. BMT nurses from the first and second years of the program will continue to be educated with the allogeneic BMT curriculum so that they will be skilled and confident in the care of patients undergoing allogeneic BMT when DMCH begins its first allogeneic transplantations.

The proposed future educational emphases of the ESNTP will include a focus on preceptorship development so that nurses at DMCH can become skilled clinical teachers and the program can become self-sustaining. Another important educational focus will be on palliative care nursing. The End-of-Life Nursing Education Consortium-International curriculum, developed by the American Association of Colleges of Nursing,^15^ will be offered as part of the ESNTP.

In conclusion, the minister of health and family welfare and other leaders within the Government of Bangladesh have fully supported this multifaceted project at every level—financially, administratively, and conceptually. For the first time, the people of Bangladesh have access to life-saving BMT. The collaborations and regard established between colleagues in Boston and in Dhaka are based on a shared vision and a shared commitment to offering excellent, accessible care.

The high levels of specialty-focused nursing practice and professional recognition achieved by nurses at DMCH who had the courage and commitment to undertake rigorous education to care for patients in Bangladesh with bone marrow malignancies and other cancers are paving the way for wider recognition of the power of excellent nursing care across Bangladesh. Nurses from Boston have deep admiration for their nurse, physician, faculty, and government leader colleagues in Bangladesh. The commitment to offering the best care possible to the people of Bangladesh, despite enormous challenges, is inspirational. Warm welcome, generous sharing, and grateful appreciation are always extended to the Boston team. The generosity, resilience, and creative problem solving of health care professionals in Bangladesh have offered much to the nurses and physicians from Boston. The teaching and learning are transformative and mutual. Future educational endeavors are being developed to continue this groundbreaking and highly rewarding collaboration.
